# Complementary Effects of Virus Population Are Required for Efficient Virus Infection

**DOI:** 10.3389/fmicb.2022.877702

**Published:** 2022-05-13

**Authors:** Yuechao Sun, Yu Zhang, Xiaobo Zhang

**Affiliations:** College of Life Sciences and Southern Marine Science and Engineering Guangdong Laboratory (Zhuhai), Zhejiang University, Hangzhou, China

**Keywords:** virus population, complementary effect, virus infection, functional gene, lncRNA

## Abstract

It is believed that the virions of a virus infecting a host may share the identical viral genome and characteristics. However, the role of genomic heterogeneity of the virions of a virus in virus infection has not been extensively explored. To address this issue, white spot syndrome virus (WSSV), a DNA virus infecting crustaceans, was characterized in the current study. In WSSV, differences in two nucleotides of the viral genome generated two types of WSSV, forming a virus population that consisted of Type A WSSV (encoding WSSV lncRNA-24) and Type B WSSV (encoding the wsv195 gene) at a ratio of 1:3. The virus populations in all virus-infected cells and tissues of different hosts exhibited a stable 1:3 structure. WSSV lncRNA-24 in Type A WSSV promoted virus infection by binding to shrimp and WSSV miRNAs, while the wsv195 gene in Type B WSSV played an essential role in virus infection. Loss of Type A WSSV or Type B WSSV in the WSSV population led to a 100-fold decrease in viral copy number in shrimp. Simultaneous loss of both types of WSSV prevented virus infection. These results indicated that the virus infection process was completed by two types of WSSV encoding different functional genes, revealing the complementary effects of WSSV population. Therefore, our study highlights the importance of the complementarity of virus population components in virus infection.

## Introduction

There are about 10^31^ virus-like particles inhabiting our planet, which outnumber all cellular life forms on the earth (Suttle, 2005; Wigington et al., 2016). Despite the presence of viruses in astonishing number and the impact of viruses on the population dynamics and evolutionary trajectories of hosts, our knowledge about the genomic properties of viruses remains limited (Mahmoudabadi and Phillips, 2018). The genomic sequence of a virus is referred to as its “genomic fingerprint,” which makes a virus unique (Lau et al., 2020). The large number of progeny (10^3^–10^4^) produced from an individual infected cell allows viruses to sample a large genetic space more rapidly than their hosts, helping them to evolve around adaptive and therapeutic defenses and to benefit from cooperative interactions among co-infecting quasispecies (Stray and Air, 2001; Chen et al., 2007). The remarkable capacity of viruses to adapt to new hosts and environments is highly dependent on their ability to generate *de novo* diversity in a short period of time (Sanjuán and Domingo-Calap, 2016). Rates of spontaneous mutations vary amply among viruses (Sanjuán and Domingo-Calap, 2016). Viral mutation rates are modulated at different levels, including polymerase fidelity, sequence context, template secondary structure, cellular microenvironment, replication mechanisms, proofreading, and access to post-replicative repair (Sanjuán, 2012). The mutations of a viral genome, which occur naturally over time (Sun et al., 2020), generate different strains of a virus. It is popularly believed that a viral strain shares the same genomic sequence.

Groups of the same-species virus that share a set of genome mutations are referred to as a lineage, which includes some strains (Verhagen et al., 2021). Some lineages may have characteristics such as the ability to spread more quickly, or to cause more severe disease (Hufsky et al., 2019). These lineages are classified as variants of interest, variants being monitored, or variants of high concern. At present, the virulence and infectivity of viral strains are often well characterized. Nevertheless, the interactions between the viral strains are generally ignored (Mostafa et al., 2018). With the spread of the Coronavirus disease 2019 (COVID-19) pandemic to almost all nooks and corners of the world, the accepted view is that there are clear differences between different strains of the COVID-19 (Goel et al., 2020; Yamamoto and Bauer, 2020; Rangayasami et al., 2021). The differences in virulence and infectivity of different strains of COVID-19 have been identified (Jeyanathan et al., 2020; Seah and Agrawal, 2020). However, the interaction between COVID-19 strains is not explored. Hepatitis B virus (HBV) is a diverse double-stranded DNA virus with 9 genotypes (A–I) and a putative 10th genotype (J), being characterized thus far (McNaughton et al., 2020). In a genotype of HBV, the viral genome is generally consistent. During virus infection, viruses must navigate the complex and unpredictable environments inside and outside the host. The genetic variability of a virus is central to virus infection, as it helps the virus evades host immunity, withstands antiviral drugs, expands the host ranges, and adopts new routes of transmission. It is a popular phenomenon that a virus has multiple different viral strains, suggesting that a virus may exist as a viral population consisting of different strains. However, the genomic heterogeneity of a virus and its role and mechanism in virus-host interaction have not been intensively explored.

To address this issue, the virus population of white spot syndrome virus (WSSV), a DNA virus infecting crustaceans (Li et al., 2019), was explored in this study. Our results revealed the existence of a WSSV population consisting of two types of WSSV, Type A WSSV (encoding WSSV lncRNA-24) and Type B WSSV (encoding the wsv195 gene). Complementary effects of the two types of WSSV were required for virus infection.

## Results

### Two Types of White Spot Syndrome Virus in the Virus Population in Infected Shrimp

**FIGURE 1:**
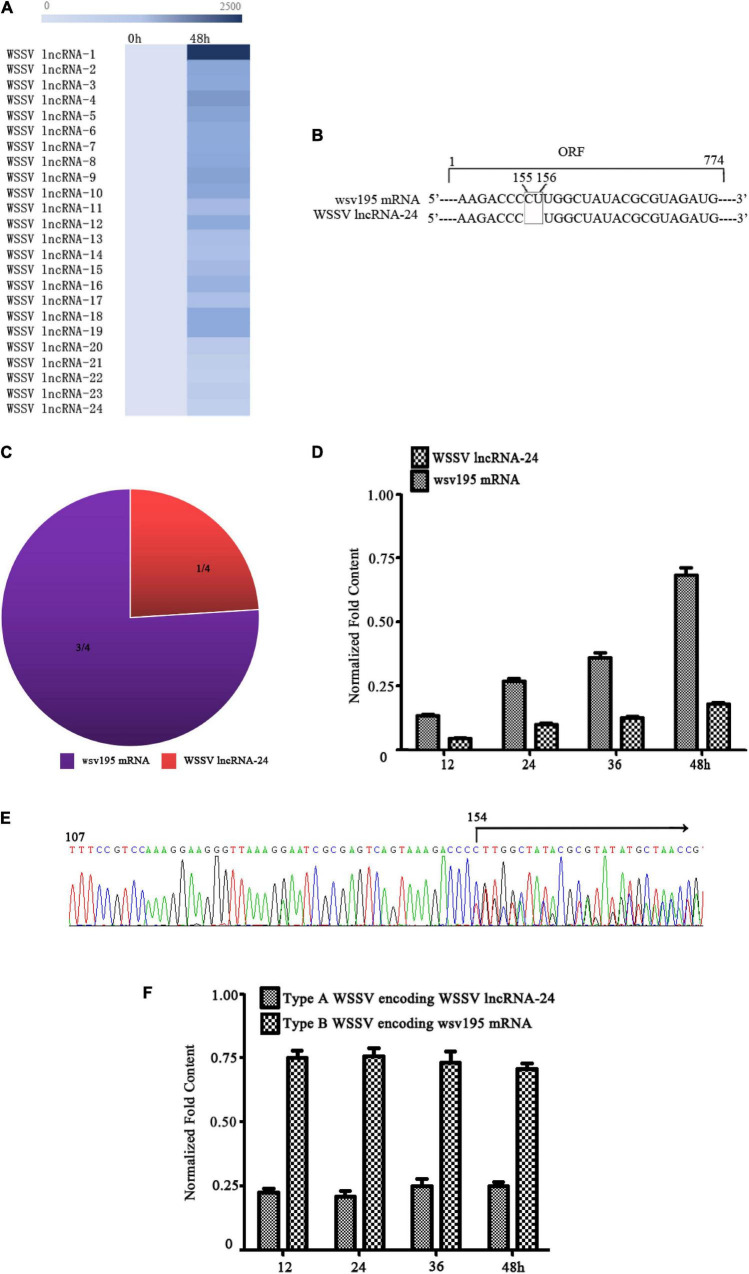
Two types of WSSV in the virus population in infected shrimp. **(A)** Heat map of WSSV lncRNA expression profiles in WSSV-challenged shrimp. The numbers indicate the time points post infection. **(B)** Sequence comparison of WSSV lncRNA-24 and wsv195 mRNA. The deletion sites are boxed. **(C)** Ratios of WSSV lncRNA-24 to wsv195 mRNA among the sequenced RNAs of WSSV-infected shrimp haemocytes. **(D)** Coexistence of WSSV lncRNA-24 and wsv195 mRNA in WSSV-infected shrimp. Shrimp were challenged with WSSV. At different time points post infection, the expression levels of WSSV lncRNA-24 and wsv195 mRNA were evaluated using quantitative real-time PCR with a specific probe differentiating wsv195 mRNA from WSSV lncRNA-24. U6 was used as the internal control. The experiment was biologically repeated for three times. **(E)** Genomic DNA sequencing of WSSV from individual virus-infected shrimp. The arrow indicates the distinguishing sites of wsv195 mRNA and WSSV lncRNA-24. **(F)** Examination of Type A WSSV encoding WSSV lncRNA-24 and Type B WSSV encoding wsv195 mRNA. The genomic DNA of WSSV was subjected to quantitative real-time PCR to detect the DNA encoding WSSV lncRNA-24 or wsv195 mRNA in WSSV-infected shrimp. The numbers indicate the times post infection. The assay was biologically repeated for three times.

To explore whether virus populations exist, WSSV, a DNA virus infecting crustaceans, was characterized. LncRNA sequencing of WSSV-infected *Marsupenaeus japonicus* haemocytes indicated that WSSV generated a total of 24 WSSV lncRNAs (Figure 1A). Among these lncRNAs, WSSV lncRNA-24 was a two-nucleotide deletion mutant of a WSSV mRNA (wsv195 mRNA) (Figure 1B). Therefore, WSSV lncRNA-24 (GenBank accession no. MN475881) was further investigated. RNA-seq data revealed that the ratio of WSSV lncRNA-24 to wsv195 mRNA contents was 1:3 (Figure 1C). To confirm the coexistence of WSSV lncRNA-24 and wsv195 mRNA, quantitative real-time PCR was conducted with a specific probe that could differentiate the two molecules. The results showed that WSSV lncRNA-24 and wsv195 mRNA coexisted in WSSV-infected shrimp at a ratio of 1:3 during virus infection (Figure 1D).

To determine whether WSSV lncRNA-24 was generated during post-transcriptional processing or existed in the viral genome, WSSV genomic DNA from individual WSSV-infected shrimp was tested for WSSV lncRNA-24 and wsv195 mRNA. The sequencing results revealed that there were two types of WSSV genomes: one encoding WSSV lncRNA-24 and another encoding wsv195 mRNA (Figure 1E), thus yielding two types of WSSV, Type A WSSV encoding WSSV lncRNA-24 and Type B WSSV encoding wsv195 mRNA. The ratio of Type A WSSV to Type B WSSV was approximately 1–3 (Figure 1F), which was consistent with the RNA-seq data. These results indicated that the change in two nucleotides of the viral genome formed a virus population consisting Type A WSSV and Type B WSSV.

Taken together, these findings showed that two types of WSSV simultaneously existed in individual WSSV-infected shrimp, suggesting that the population of the two types of WSSV was required for the WSSV infection.

### Existence of Virus Populations in Situations Ranging From Single Virus-Infected Cells to Different Virus-Infected Hosts

**FIGURE 2:**
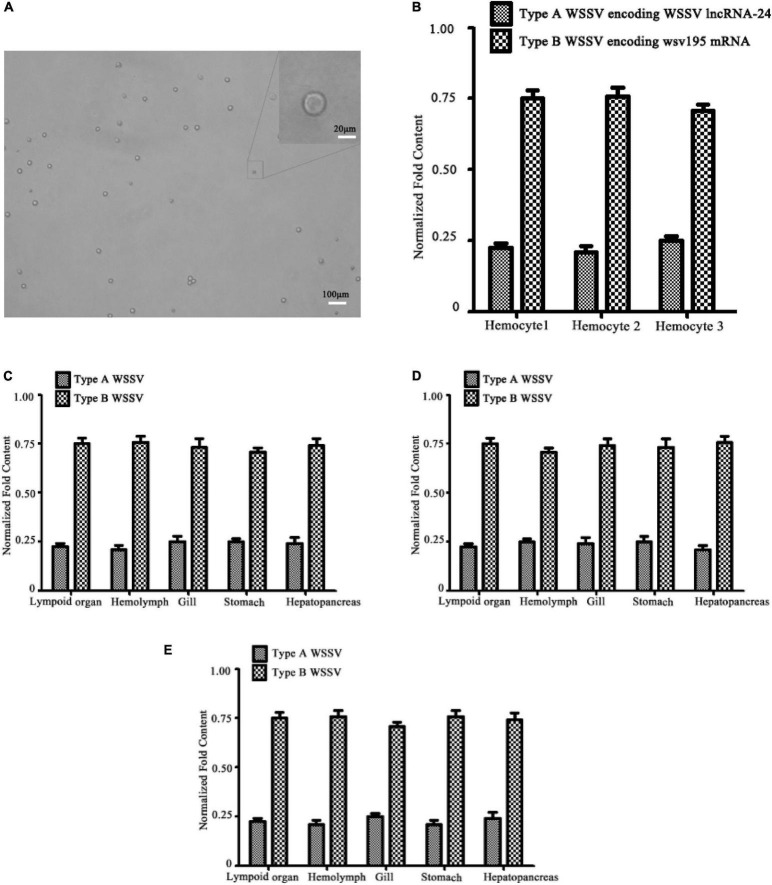
Existence of virus populations in situations ranging from single virus-infected cells to different virus-infected hosts. **(A)** Isolation of single haemocytes from the haemolymph of WSSV-infected shrimp. **(B)** Existence of two types of WSSV genomes in single shrimp haemocytes. Single haemocytes from the haemolymph of WSSV-infected shrimp were subjected to quantitative real-time PCR to detect Type A WSSV and Type B WSSV. **(C)** Identification of viral genome heterogeneity in different tissues or organs of WSSV-infected shrimp. Shrimp were infected with WSSV. At 48 h post infection, the WSSV genome in different tissues or organs of shrimp was detected with quantitative real-time PCR. **(D)** Viral genome heterogeneity in different tissues or organs of WSSV-infected crayfish. **(E)** Distribution of two types of WSSV genomes in different tissues or organs of WSSV-infected mud crabs. All the experiments are biologically repeated for three times.

To investigate whether the WSSV population containing Type A WSSV and Type B WSSV existed in single haemocytes, the single haemocytes were isolated from WSSV-infected shrimp haemolymph (Figure 2A). The results indicated that there were indeed two types of WSSV virions encoding WSSV lncRNA-24 (Type A WSSV) and wsv195 mRNA (Type B WSSV) in a single shrimp haemocyte (Figure 2B). The ratio of Type A WSSV to Type B WSSV was 1:3 (Figure 2B). These data confirmed that Type A WSSV and Type B WSSV formed a WSSV population in a single shrimp haemocyte. Viruses complete their life cycles in host cells. Therefore, our results suggest that virus populations in which the virions contain different genomes to execute different functions facilitate virus infection.

To explore the distribution of the WSSV population in different tissues or organs, the presence of Type A WSSV or Type B WSSV was examined in the shrimp lymphoid organ, haemolymph, gills, stomach and hepatopancreas, which can be infected by WSSV. The results showed that the types of WSSV existed in all examined tissues or organs at a ratio of 1:3 (Type A WSSV : Type B WSSV) (Figure 2C). The structures of the WSSV populations in different shrimp tissues and organs were identical to that in single shrimp haemocytes, indicating that the virus population consistently formed the stable 1:3 structure.

To further characterize the structure of the WSSV population in different hosts, Type A WSSV and Type B WSSV were examined in crayfish (*Procambarus clarkii*) and mud crabs (*Scylla paramamosain*). The results again revealed a 1:3 ratio (Type A WSSV : Type B WSSV) in different tissues or organs of virus-infected crayfish (Figure 2D). The analysis of the mud crabs generated essentially the same results (Figure 2E). These data showed that the structure of the WSSV population remained stable in different hosts.

Taken together, the above findings showed that the virus population existed in all virus-infected cells and tissues of different hosts and that its structure was stable with changing external stimuli.

**FIGURE 3:**
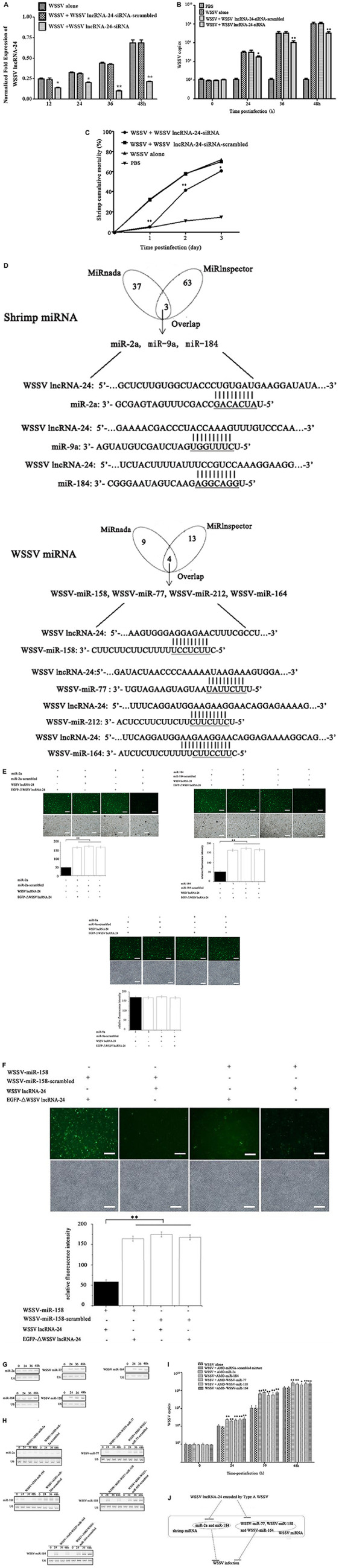
Influence of Type A WSSV in the virus population on virus infection. **(A)** Silencing of WSSV lncRNA-24 encoded by Type A WSSV in WSSV-infected shrimp. Sequence-specific siRNA targeting WSSV lncRNA-24 and WSSV were coinjected into shrimp to knock down WSSV lncRNA-24. WSSV lncRNA-24-siRNA-scrambled was included in the injection as a control. At different time points post infection, the expression level of WSSV lncRNA-24 was examined using quantitative real-time PCR. **(B)** Effects of WSSV lncRNA-24 silencing on virus replication in shrimp. The WSSV copy numbers in shrimp treated with WSSV lncRNA-24-siRNA were quantified by real-time PCR. PBS, WSSV alone and WSSV lncRNA-24-siRNA-scrambled were used as controls. **(C)** Influence of WSSV lncRNA-24 silencing on shrimp mortality. The mortality of shrimp treated with WSSV lncRNA-24-siRNA was examined every day. The treatments are indicated at the top. **(D)** Prediction of miRNAs targeted by WSSV lncRNA-24. The seed sequences of miRNAs are underlined. **(E)** Direct interaction between WSSV lncRNA-24 and shrimp miR-2a, miR-9a or miR-184. Insect High Five cells were cotransfected with miR-2a, miR-9a or miR-184 and the plasmid EGFP-WSSV lncRNA-24 or EGFP-ΔWSSV lncRNA-24. At 36 h after cotransfection, the fluorescence intensity of insect cells was evaluated. Scale bar, 50 μm. **(F)** Interaction between WSSV lncRNA-24 and WSSV miRNAs. Insect High Five cells were cotransfected with a WSSV miRNA (WSSV-miR-158, WSSV-miR-77, WSSV-miR-212, or WSSV-miR-164) and the plasmid EGFP-WSSV lncRNA-24 or EGFP-ΔWSSV lncRNA-24. At 36 h after cotransfection, the fluorescence intensity of insect cells was examined. Scale bar, 50 μm. **(G)** Expression levels of miR-2a, miR-184, WSSV miR-77, WSSV miR-158, and WSSV miR-164 in virus-infected shrimp. Shrimp were challenged with WSSV. At different times post infection, the expression of miR-2a, miR-184, WSSV miR-77, WSSV miR-158, and WSSV miR-164 was detected in the haemocytes of virus-infected shrimp by Northern blot analysis. U6 was used as a control. The probes are indicated on the left. **(H)** Silencing of shrimp or WSSV miRNAs in WSSV-infected shrimp. Shrimp were coinjected with WSSV and AMO-miR-2a, AMO-miR-184, AMO-WSSV-miR-77, AMO-WSSV-miR-158, or AMO-WSSV-miR-164. AMO-miRNA-scrambled was included in the assays as a control. At different times post infection, the miRNA was detected by Northern blot analysis. The probes used are indicated on the left. The numbers show the time points post infection. U6 was used as a control. **(I)** Effects of shrimp or WSSV miRNA silencing on virus infection. WSSV and AMO-miRNA were coinjected into shrimp. A mixture of AMO-miR-2a-scrambled, AMO-miR-184-scrambled, AMO-WSSV-miR-77-scrambled, AMO-WSSV-miR-158- scrambled and AMO-WSSV-miR-164-scrambled was used as a control. At different times after coinjection, the WSSV copies were examined with quantitative real-time PCR. **(J)** Model for the role of Type A WSSV in virus infection. All the assays were biologically repeated for three times. In all panels, the statistical significance between treatments is indicated with asterisks (*p < 0.05; **p < 0.01).

### Influence of Type A White Spot Syndrome Virus in the Virus Population on Virus Infection

To elucidate the role of Type A WSSV encoding WSSV lncRNA-24 in virus infection, the expression of WSSV lncRNA-24 was knocked down via injection of a sequence-specific siRNA (WSSV lncRNA-24-siRNA) into shrimp *in vivo* followed by evaluation of virus infection. The results indicated that WSSV lncRNA-24 was successfully silenced by WSSV lncRNA-24-siRNA in WSSV-infected shrimp (Figure 3A). The silencing significantly decreased the WSSV copy numbers (Figure 3B) and virus-infected shrimp mortality (Figure 3C) compared to those of the controls. The data showed that WSSV lncRNA-24 encoded by Type A WSSV exerted a positive effect on WSSV replication.

To explore the underlying mechanism of WSSV lncRNA-24, the protein and DNA molecules that interact with WSSV lncRNA-24 were screened, but no binding protein or DNA molecules were found. Thus, the miRNAs interacting with WSSV lncRNA-24 were characterized.

The prediction analysis showed that WSSV lncRNA-24 can target shrimp miRNAs (miR-2a, miR-9a and miR-184) and WSSV miRNAs (WSSV-miR-77, WSSV-miR-158, WSSV-miR-164, and WSSV-miR-212) (Figure 3D). To evaluate the interactions between WSSV lncRNA-24 and shrimp or WSSV miRNAs, the plasmid pIZ/EGFP-WSSV lncRNA-24 containing EGFP and WSSV lncRNA-24 was cotransfected with a miRNA into insect cells. The results indicated that the fluorescence intensity of the cells cotransfected with shrimp miR-2a or miR-184 and pIZ/EGFP-WSSV lncRNA-24 was significantly lower than that of the controls (Figure 3E). However, the fluorescence intensity of the cells cotransfected with miR-2a or miR-184 and EGFP-ΔWSSV lncRNA-24 was similar to that of the controls (Figure 3E). Treatment with shrimp miR-9a did not change the fluorescence intensity of the cells (Figure 3E). These data revealed that WSSV lncRNA-24 interacted with shrimp miR-2a and miR-184.

To characterize the interactions between WSSV lncRNA-24 and WSSV miRNAs, WSSV lncRNA-24 was cotransfected with WSSV-miR-77, WSSV-miR-158, WSSV-miR-164 or WSSV-miR-212 into insect cells. The data showed that WSSV lncRNA-24 interacted with WSSV-miR-77, WSSV-miR-158 and WSSV-miR-164 (Figure 3F).

To reveal the roles of miRNA interactions with WSSV lncRNA-24, the expression levels of miRNAs in the haemocytes of WSSV-infected shrimp were examined. Northern blots indicated that shrimp miR-2a, shrimp miR-184, WSSV-miR-77, WSSV-miR-158, and WSSV-miR-164 were significantly upregulated in shrimp in response to WSSV infection (Figure 3G), suggesting that these miRNAs were involved in the infection process. When the expression of miR-2a, miR-184, WSSV-miR-77, WSSV-miR-158, or WSSV-miR-164 was knocked down with a sequence-specific siRNA in WSSV-infected shrimp (Figure 3H), the WSSV copy numbers were significantly higher than those in the control group (Figure 3I), indicating the negative roles of shrimp miR-2a, shrimp miR-184, WSSV-miR-77, WSSV-miR-158, and WSSV-miR-164 in virus infection.

Taken together, the findings revealed that Type A WSSV in the virus population promoted virus infection by encoding WSSV lncRNA-24 to suppress the expressions of shrimp and WSSV miRNAs (Figure 3J).

### Impact of Type B White Spot Syndrome Virus in the Virus Population on Virus Infection

**FIGURE 4:**
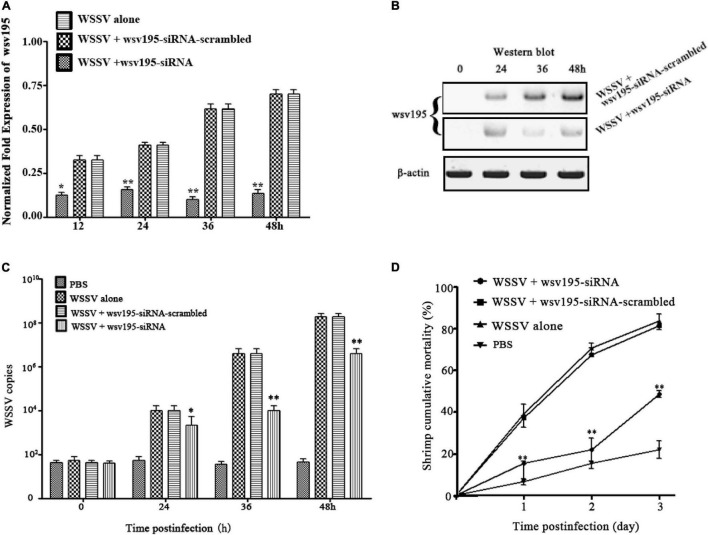
Impact of Type B WSSV in the virus population on virus infection. **(A)** Silencing of wsv195 encoded by Type B WSSV in WSSV-infected shrimp. Wsv195-siRNA and WSSV were coinjected into shrimp. As a control, wsv195-siRNA-scrambled was included in the assay. At different time points post infection, the wsv195 mRNA level was examined using quantitative real-time PCR. The number indicates the time post infection. **(B)** Western blot analysis of wsv195-silenced shrimp. The treatments are indicated on the right. β-Actin was used as a control. **(C)** Effects of wsv195 silencing on virus replication in shrimp. The WSSV copy numbers in shrimp treated with wsv195-siRNA were quantified by real-time PCR. PBS, WSSV alone and wsv195-siRNA-scrambled were used as controls. **(D)** Influence of wsv195 knockdown on WSSV-infected shrimp mortality. All the experiments were biologically repeated for three times. In all panels, the statistical significance between treatments is indicated with asterisks (*p < 0.05; **p < 0.01).

To elucidate the role of Type B WSSV encoding wsv195 mRNA in virus infection, the expression of the wsv195 gene was knocked down by injecting a sequence-specific siRNA (wsv195-siRNA) into shrimp. The results confirmed that the expression of wsv195 was silenced by wsv195-siRNA in WSSV-infected shrimp compared with controls (Figure 4A). Western blot analysis generated similar results (Figure 4B).

The results showed that silencing wsv195 expression significantly decreased the WSSV copy numbers in WSSV-infected shrimp compared with controls (Figure 4C). In addition, wsv195 silencing led to a significant decrease in WSSV-infected shrimp mortality (Figure 4D). These findings demonstrated that Type B WSSV exerted a positive effect on WSSV replication in shrimp via encoding wsv195 protein.

### Complementary Effects of Type A White Spot Syndrome Virus and Type B White Spot Syndrome Virus in the Virus Population on Virus Infection

**FIGURE 5:**
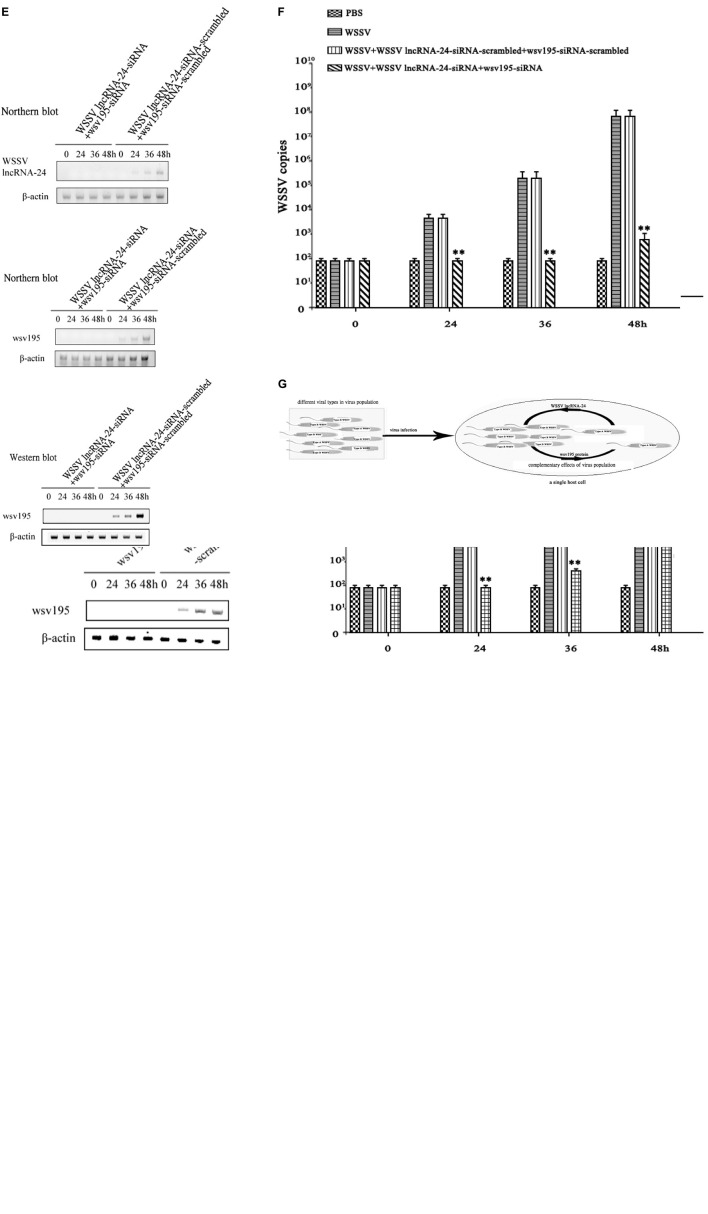
Complementary effects of Type A WSSV and Type B WSSV in the virus population on virus infection. **(A)** Knockdown of WSSV lncRNA-24 encoded by Type A WSSV in shrimp. Shrimp were injected with WSSV lncRNA-24-siRNA or WSSV lncRNA-24-siRNA-scrambled. Twelve hours later, the shrimp were coinjected with siRNA and WSSV and then with siRNA three times at 12-h intervals. At different times after the WSSV injection, the expression levels of WSSV lncRNA-24 in shrimp haemocytes were examined by Northern blot analysis. β-actin was used as a control. **(B)** Influence of Type A WSSV on WSSV content in shrimp. Shrimp were injected with WSSV and WSSV lncRNA-24-siRNA. At different times post infection, the WSSV copy numbers in shrimp haemocytes were determined by quantitative real-time PCR. PBS, WSSV, and siRNA-scrambled were included in the assays as controls. The statistical significance between treatments is indicated with asterisks (^**^p < 0.01). **(C)** Silencing of wsv195 in shrimp. Shrimp were injected with wsv195-siRNA or wsv195-siRNA-scrambled. Twelve hours later, the shrimp were coinjected with siRNA (wsv195-siRNA or wsv195-siRNA-scrambled) and WSSV and then with the siRNA three times at 12-h intervals. At different times after the last injection, the expression level of WSSV lncRNA-24 in shrimp haemocytes was examined by Northern blot analysis (top) and Western blot analysis (bottom). Shrimp β-actin was used as a control. **(D)** Role of Type B WSSV in virus infection. Shrimp were injected with WSSV and WSSV wsv195-siRNA. At different times post infection, the WSSV copies in shrimp hemocytes were determined by quantitative real-time PCR. PBS, WSSV and siRNA-scrambled were used as controls. The statistical significance between treatments is indicated with asterisks (^**^p < 0.01). **(E)** Simultaneous depletion of Type A WSSV and Type B WSSV in WSSV-infected shrimp. Shrimp were injected with WSSV lncRNA-24-siRNA and wsv195-siRNA. Twelve hours later, the shrimp were co-injected with siRNAs (WSSV lncRNA-24-siRNA and wsv195-siRNA) and WSSV and then with the siRNAs three times at 12-h intervals. WSSV lncRNA-24-siRNA-scrambled and wsv195-siRNA-scrambled were included in the injection as controls. At different times after the last injection, the expression levels of WSSV lncRNA and wsv195 in shrimp haemocytes were examined by Northern blot and Western blot. Shrimp β-actin was used as a control. **(F)** Impact of simultaneous loss of Type A WSSV and Type B WSSV on virus infection in shrimp. Shrimp were co-injected with wsv195-siRNA, WSSV lncRNA-24 and WSSV. The WSSV content in shrimp was examined at different time after injection. The statistical significance between treatments is indicated with asterisks (^**^p < 0.01). **(G)** Model for the complementary effects of virus population during virus infection. All the experiments were biologically repeated for three times.

To further evaluate the role of the virus population in virus infection, the expression of WSSV lncRNA-24 encoded by Type A WSSV or/and the wsv195 gene encoded by Type B WSSV was silenced in WSSV-infected shrimp before examination of virus infection. Northern blots indicated that the expression of WSSV lncRNA-24 was completely silenced in the WSSV-infected shrimp (Figure 5A). The results showed that the depletion of Type A WSSV led to significant decreases of virus copies in shrimp (Figure 5B). At 48 h post-infection, the WSSV content in shrimp without Type A WSSV was 100-fold lower than that in the positive controls (WSSV) (Figure 5B). These data demonstrated the importance of Type A WSSV of virus population in virus infection.

To reveal the impact of Type B WSSV in virus population on virus infection, the expression of wsv195 was silenced in WSSV-infected shrimp. When Type B WSSV was completely depleted (Figure 5C), the WSSV copy numbers in shrimp hemocytes were significantly lower than those in the positive controls (infected with WSSV) (Figure 5D). At 48 h post-infection, the depletion of Type B WSSV led to a 100-fold decrease of WSSV copies in shrimp compared with the positive controls (Figure 5D), showing the importance of Type B WSSV for WSSV infection.

To further explore the impact of the simultaneous loss of Type A and Type B WSSVs on virus infection, the expressions of WSSV lncRNA-24 and wsv195 were simultaneously completely suppressed in shrimp (Figure 5E). The WSSV content in shrimp injected with WSSV lncRNA-24-siRNA and wsv195 mRNA was comparable with that in the negative controls (Figures 5E,F), showing that the simultaneous loss of Type A WSSV and Type B WSSV led to no virus infection in shrimp.

The above data demonstrated that the virus infection process was completed by different types of virus encoding different functional genes that exerted complementary effects in the virus population (Figure 5G).

## Discussion

Viruses have very small genomes, limiting the genes needed to complete their life cycle in host cells (Casey et al., 2018; Bao et al., 2019). In order to navigate complex and unpredictable environments inside and outside the host, viruses must make full use of their viral genomes. Genetic variability within a viral genome is central for virus life cycle, helping the virus evade host immunity (Dingens et al., 2017; Schroeder et al., 2017), withstand antiviral drugs (Belshe et al., 1988), expand host range (Ma et al., 2016), and adopt new routes of transmission (Imai et al., 2012). It is well known that a virus can proliferate only in its host cells. In the process of virus infection, therefore, it can be inferred that a predictable result of genetic variability of a virus in single cells should be viral genome heterogeneity. Up to date, however, whether a virus functions as a virus population in single host cells and single hosts has not been extensively explored. In this study, the findings revealed that the differences in two nucleotides of the viral genome yielded two types of WSSV, forming a virus population that consisted of Type A WSSV and Type B WSSV at a ratio of 1:3. The structure of the WSSV populations in all virus-infected cells and tissues of different hosts were stable, indicating the importance of virus population in the process of virus invasion to its hosts. In the clinic, viruses are widely known to consist of a variety of viral strains with different viral genomes, suggesting the existence of virus populations. Therefore the concept of virus populations might be universal in animals for the efficient infection of viruses, which was an efficient strategy for a virus to make full use of the viral genome during the infection process.

In this study, the results demonstrated that the loss of Type A WSSV or Type B WSSV in the WSSV population resulted in a 100-fold decrease of viral copies in hosts. When both types of WSSV in the virus population were simultaneously depleted, the virus was not able to infect its hosts. Therefore there was a complementary effect between different viral types in the WSSV population, resulting from different functional genes encoded by different viral types. In the process of virus infection, the virions with varied viral genomes proliferated in the same host cells, thus benefiting from cooperative interactions among co-infecting virions with heterogeneous viral genomes. Every type of virions could function as a helper virus to the other types of virions of the virus based on the differences in their viral genomic sequences. The existence of virus population, which allowed permissive genomic mutations to reside in different virions, was indispensable for the propagation of the virus. The consequences of the complementary effect of virus population discussed here would not be exclusive to WSSV, raising the possibility that similar considerations existed in all viruses as well. Interestingly, pleomorphism is common among enveloped viruses, with filamentous morphology common in the filoviridae, pneumoviridae, and paramyxoviridae families (Pierson and Diamond, 2012). More generally, the size, composition, and epitopes displayed on the surface of many viruses influence how they gain entry into a cell and interact with the innate and adaptive immune response (Feng and Gao, 2007; Barba-Spaeth et al., 2016), suggesting that the complementary effects in these properties could influence replicative fitness or viral tropism. The complementary effects of virus population could contribute to viral persistence in a wide variety of viruses. The more complementary the virions of a virus with heterogeneous viral genomes are, the more virulent the virus becomes. Since therapeutic interventions against virus can alter viral phenotypes in genetically independent ways, direct characterization of virus populations in the manner described here may help in evaluating the impact of emerging antiviral therapies (Furuta et al., 2013; Strauch et al., 2017). Overall, understanding the extent to which complementary effects of virus population contributes to viral persistence can help to guide the development of a new class of therapies aimed at reducing natural variability or applying combinatorial pressure that mitigates the survival benefits of phenotypic variability.

## Materials and Methods

### Animal Culture and White Spot Syndrome Virus Infection

Shrimp (*Marsupenaeus japonicus*) about 10 g/shrimp, crayfish (*Procambarus clarkii*) about 20 g/crayfish and mud crab (*Scylla paramamosain*) about 50 g/crab were cultured in tanks containing 80 liters of aerated seawater at room temperature (25°C), respectively. Based on PCR detection using WSSV-specific primers (5′-TATTGTCTC TCCTGACGTAC-3′ and 5′-CACATTCTTCACGAGTCTAC-3′), shrimp, crayfish and crab were WSSV-free prior to experimental infection. The virus-free shrimp, crayfish and crab were infected with WSSV (10^5^ virus copies/ml) by injection at 100 μl WSSV/animal, respectively. At different times postinfection, three shrimp, crayfishes or crabs were randomly collected for each treatment. The hemocytes of shrimp, crayfishes or crabs were mixed for later use.

### RNA-Seq and Data Analysis

Total RNAs were extracted from WSSV-infected shrimp hemocytes using Trizol reagent (Invitrogen, CA, United States) following the manufacturer’s procedure. The extracted RNAs were subjected to RNA-seq and then analyzed by LC Sciences (Houston, TX, United States). The sequences were mapped to the WSSV genome to find lncRNAs of WSSV. To predict WSSV lncRNAs, two independent computational algorithms CNCI^1^ and CPC (coding potential calculator) were used (Kang et al., 2017; Luo et al., 2017).

### Detection of wsv195 mRNA, White Spot Syndrome Virus lncRNA-24 and Their Corresponding DNAs by Quantitative Real-Time PCR

Total RNAs were extracted from WSSV-infected shrimp using RNAprep Pure Tissue Kit (Tiangen Biotech, Beijing, China) according to the manufacturer’s instructions. The first strand of cDNA was synthesized using PrimeScript ^®^ 1st Strand cDNA Synthesis Kit (Takara, Japan) with Oligo dT as the primer. U6 was used as a control. The expression levels of WSSV lncRNA-24 and wsv195 mRNA were examined using quantitative real-time PCR with specific primers (5′-GGAAGGGTTA AAGAAATCG-3′ and 5′-CTCGTTCCTCTTCATTCA-3′) and sequence-specific probe (WSSV lncRNA-24, 5′-CAGTAAAGACCCTGGCTATACGC-3′; wsv195 mRNA, 5′-CAGTAAAGACCCCTTGGCTATACG-3′). Quantitative real-time PCR was performed using a 2 × SYBR Premix Ex Taq Kit (Takara, Japan) according to the manufacturer’s manual. PCR was conducted at 95°C for 3 min, followed by 40 cycles at 95°C for 15 s and 60°C for 30 s.

For examination of WSSV encoding WSSV lncRNA-24 or wsv195 mRNA, the WSSV genome was extracted from virus-infected shrimp using an SQ Tissue DNA Kit (Omega Bio-Tek, United States). The viral genomic DNA was subjected to quantitative real-time PCR as described above.

### RNA Interference Assay in Shrimp *in vivo*

To silence gene expression, siRNA specifically targeting a gene was synthesized according to the design rule for siRNA with a commercial kit according to the manufacturer’s instructions (TaKaRa, Japan). The siRNAs used were WSSV lncRNA-24 siRNA (5′-CCTGAAGGAGGAGACCTAT-3′) and wsv195 siRNA (5′-G CGGCGACAGAGACTCATT-3′). As controls, the siRNA sequences were scrambled, generating WSSV lncRNA-24-siRNA-scrambled (5′-GGCGGTACGCACGAATTAA -3′) and wsv195-siRNA-scrambled (5′-GCGCAGACGCGACGTATA-3′). RNA Interference (RNAi) assay in shrimp was conducted by injecting siRNA (30 μg/shrimp) into the lateral area of the fourth abdominal segment of shrimp using a 1-ml sterile syringe. The siRNA (15 μg) and WSSV (10^5^ copies/ml) were co-injected into virus-free shrimp at 100 μl/shrimp. At 12 h after co-injection, the siRNA (15 μg) was injected into the same shrimp (100 μl/shrimp). WSSV alone (10^5^ copies/ml) and phosphate buffered saline (PBS) were included in injections as controls. For each treatment, 20 shrimps were used. At different time after injection, the shrimp were collected for later use. The assays were biologically repeated three times.

### Detection of White Spot Syndrome Virus Copies With Quantitative Real-Time PCR

To quantify WSSV copies in shrimp, the WSSV genome was extracted from virus-infected shrimp using an SQ Tissue DNA Kit (Omega Bio-Tek, United States) and then subjected to quantitative real-time PCR using WSSV-specific primers (5′-TTGGTTT CAGCCCGAGATT-3′ and 5′-CCTTGGTCAGCCCCTTGA-3′) and TaqMan probe (5′-FAM-TGCTGCCGTCTCCAA-TAMRA-3′). A linearized plasmid with a 1,400-bp DNA fragment of WSSV genome was used as an internal standard for quantitative real-time PCR. PCR was conducted at 95°C for 1 min, followed by 45 cycles at 95°C for 30 s, 52°C for 30 s, and 72°C for 30 s.

### Western Blot Analysis

Proteins were separated by 12% SDS-polyacrylamide gel electrophoresis and then transferred onto a nitrocellulose membrane. After blocking with 5% non-fat milk in TBST (10 mM Tris-HCl, 150 mM NaCl, 20% Tween 20, pH7.5) for 2 h at room temperature, the membrane was incubated with a primary antibody overnight, followed by incubation with horseradish peroxidase-conjugated secondary antibody (Bio-Rad, United States) for 2 h at room temperature. The primary antibodies were prepared in our laboratory. Subsequently the membrane was detected using a Western Lightning Plus-ECL kit (Perkin Elmer, United States).

### Interactions Between White Spot Syndrome Virus lncRNA-24 and miRNAs

Insect High Five cells (Invitrogen, United States) were cultured at 27°C in Express Five serum-free medium (Invitrogen) containing l-glutamine (Invitrogen). The cells at about 70% confluence were co-transfected with a synthesized WSSV or shrimp miRNA (300 nM) and a plasmid consisting of the EGFP gene and WSSV lncRNA-24. The miRNAs were synthesized with the D6140 *in vitro* transcription T7 kit (TaKaRa, Japan). All transfections were carried out in triplicate with Cellfectin transfection reagent (Invitrogen) according to the manufacturer’s protocol. At 48 h after transfection, the fluorescence of cells was examined with a Flex Station II microplate reader (Molecular Devices, United States) at 490 and 510 nm for excitation and emission, respectively. The fluorescence values were corrected by subtracting the auto fluorescence of cells not expressing EGFP. All the experiments were repeated biologically three times.

### Detection of miRNAs by Northern Blotting

Total RNAs were extracted from shrimp hemocytes with mirVana miRNA isolation kit (Ambion, United States). After separation on a denaturing 15% polyacrylamide gel containing 7 M urea, the RNAs were transferred to a Hybond-N+ nylon membrane, followed by ultraviolet cross-linking. The membrane was prehybridized in DIG (digoxigenin) Easy Hyb granule buffer (Roche, Switzerland) for 0.5 h at 42°C and then hybridized with a DIG-labeled miRNA (miR-2a, 5′-TATCACAGCCAGCTTTG ATGAGCG-3′; miR-184, 5′-UGGACGGAGAACUGAUAAGGGC-3′; WSSV-miR- 77, 5′-TTTCTTATAATGATGAAGATGT-3′; WSSV-miR-158, 5′-CTTCTCCTTTT TCTTCTTCTTC-3′; WSSV-miR-158, 5′-CTTCCTTCTTTTTCTTCTCTA-3′; U6, 5′ -GGGCCATGCTAATCTTCTCTGTATCGTT-3′) at 42°C overnight. Subsequently the detection was performed with the DIG High Prime DNA labeling and detection starter kit II (Roche).

### Silencing of miRNA in Shrimp

To knock down the expression of miR-2a, miR-184, WSSV-miR-77, WSSV-miR-158 or WSSV-miR-164 in shrimp, a sequence-specific anti-miRNA oligonucleotide (AMO) was injected into WSSV-infected shrimp. AMO-miR-2a (5′-CGCTCATCAAAGCTGGCTGTGATA-3′), AMO-miR-184 (5′-GCCCTTATCA GTTCTCCGTCCA-3′), AMO-WSSV-miR-77 (5′-ACATCTTCATCATTATAAGAA A-3′), AMO-WSSV-miR-158 (5′-GAAGAAGAAGAAAAAGGAGAAG-3′) and AMO-WSSV-miR-164 (5′-TAGAGAAGAA AAAGAAGGAAG-3′) were synthesized (Sangon Biotech, Shanghai, China) with a phosphorothioate backbone and a 2′-O-methyl modification at the 12th nucleotide. AMO (10 nM) and WSSV (10^5^ copies/ml) were co-injected into virus-free shrimp at a 100 μl/shrimp. At 12 h after the co-injection, AMO (10 nM) was injected into the same shrimp. As controls, AMO-miR -2a-scrambled (5′-TTGCATGTCTGTCGAG-3′), AMO-miR-184-scrambled (5′-TTG CATGTCTGTCGAG-3′), AMO-WSSV-miR-77-scrambled (5′-TTGCATGTCTGTC GAG-3′), AMO-WSSV-miR-158-scrambled (5′-TTGCATGTCTGTCGAG-3′), AMO-WSSV-miR-164-scrambled (5′-TTGCATGTCTGTCGAG-3′), WSSV alone (10^5^ copies/ml) and phosphate buffered saline (PBS) were included in the injections. At different time after injection, the shrimp hemocytes were collected for later use.

### Statistical Analysis

To calculate the mean and standard deviation, the numerical data from three independent experiments were analyzed by one-way analysis of variance (ANOVA). The differences between treatments were analyzed by *t*-test.

## Data Availability Statement

The data presented in the study are deposited in the NCBI repository, accession number SRR18218566.

## Author Contributions

XZ and YS conceptualized the study and designed the experiments. YS and YZ performed the experiments. XZ and YS wrote the manuscript with contributions from all authors.

## Conflict of Interest

The authors declare that the research was conducted in the absence of any commercial or financial relationships that could be construed as a potential conflict of interest.

## Publisher’s Note

All claims expressed in this article are solely those of the authors and do not necessarily represent those of their affiliated organizations, or those of the publisher, the editors and the reviewers. Any product that may be evaluated in this article, or claim that may be made by its manufacturer, is not guaranteed or endorsed by the publisher.

## Footnotes

1
https://github.com/www-bioinfo-org/CNCI
