# Corrigendum: Complementary effects of virus population are required for efficient virus infection

**DOI:** 10.3389/fmicb.2025.1535353

**Published:** 2025-03-06

**Authors:** Yuechao Sun, Yu Zhang, Xiaobo Zhang

**Affiliations:** College of Life Sciences and Southern Marine Science and Engineering Guangdong Laboratory (Zhuhai), Zhejiang University, Hangzhou, China

**Keywords:** virus population, complementary effect, virus infection, functional gene, lncRNA

In the published article, there was an error in Figure 3F as published. The images of “WSSV-miR-158+ EGFP-ΔWSSV lncRNA-24” and “WSSV-miR-158-scrambled +WSSV lncRNA-24” in Figure 3F were inadvertently used. The corrected Figure 3F and its caption appear below.

**Figure 3:**
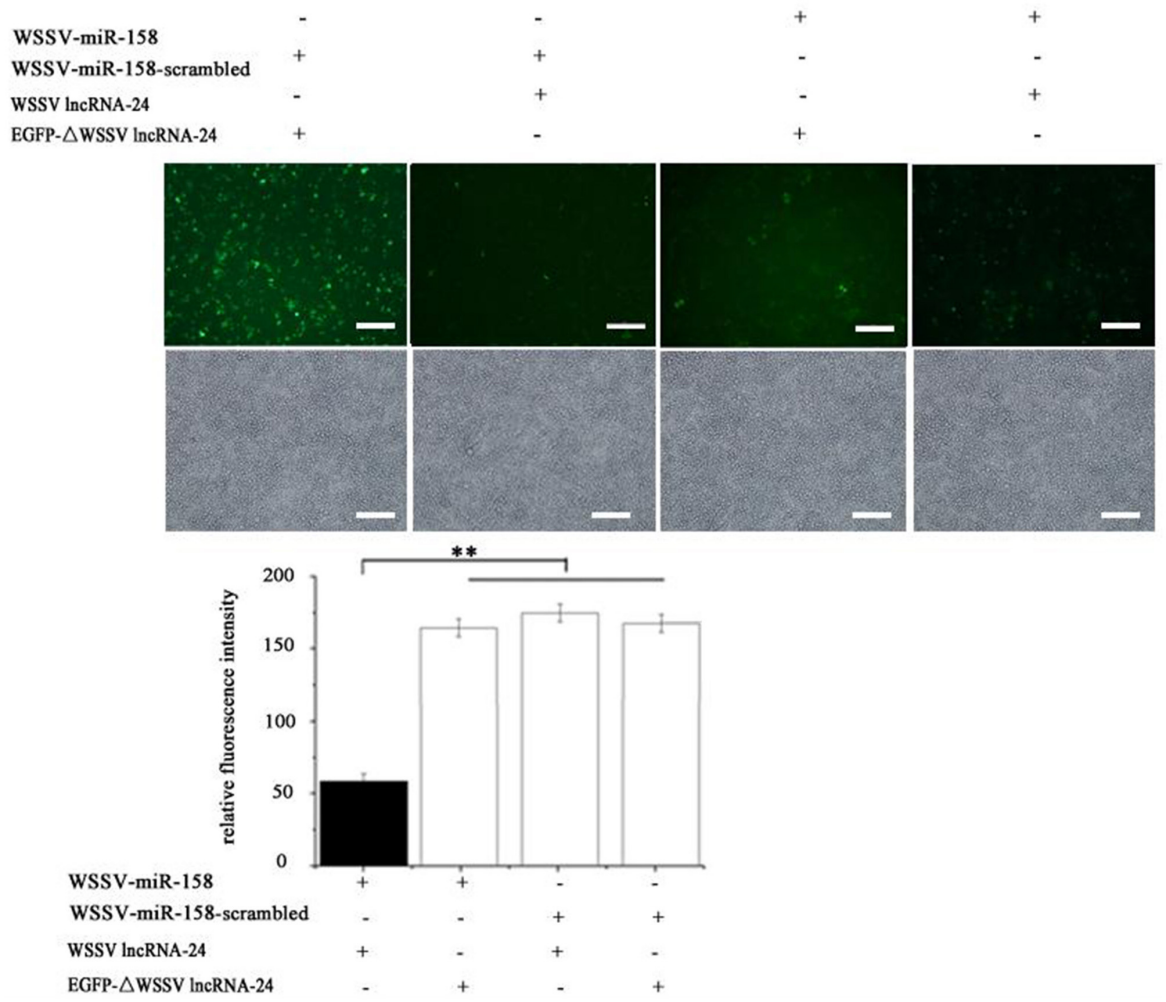
**(F)** Interaction between WSSV lncRNA-24 and WSSV miRNAs. Insect High Five cells were cotransfected with a WSSV miRNA (WSSV-miR-158, WSSV-miR-77, WSSV-miR-212, or WSSV-miR-164) and the plasmid EGFP-WSSV lncRNA-24 or EGFP-ΔWSSV lncRNA-24. At 36 h after cotransfection, the fluorescence intensity of insect cells was examined. Scale bar, 50 μm.

The authors apologize for this error and state that this does not change the scientific conclusions of the article in any way. The original article has been updated.

